# Supplementary material for: Low level SARS-CoV-2 RNA detected in plasma samples from a cohort of Nigerians: Implications for blood transfusion
Source: PLoS One. 2021 Jun 10;16(6):e0252611. doi: 10.1371/journal.pone.0252611 (PMC8191972; doi:10.1371/journal.pone.0252611)
Supplement: Attachment — Submitted filename: Second review_Low-level SARS-CoV-2 RNA detected in Plasma Samples from a cohort of Nigerians.docx [file pone.0252611.s002.docx]

Low level SARS-CoV-2 RNA detected in plasma samples from a cohort of Nigerians: Implications for blood transfusion

The authors have addressed most of my questions. Only two minor corrections need to be done:

1. Please, include the starting amount of plasma (200 ul) for RNA extraction is the Methods section.
2. Please, include the statistic test that was applied (Chi-square, Fisher exact test, etc) in Methods and in Results sections.
