# Supplementary material for: Low level SARS-CoV-2 RNA detected in plasma samples from a cohort of Nigerians: Implications for blood transfusion
Source: PLoS One. 2021 Jun 10;16(6):e0252611. doi: 10.1371/journal.pone.0252611 (PMC8191972; doi:10.1371/journal.pone.0252611)
Supplement: Attachment — Submitted filename: Response to reviewers PLOS ONE.docx [file pone.0252611.s003.docx]

**Response to Reviewers**

**Please note: My responses are in bold**

PONE-D-20-37710R1
Low-level SARS-CoV-2 RNA detected in Plasma Samples from a cohort of Nigerians: implications for blood transfusion
PLOS ONE

1. Please ensure that your manuscript meets PLOS ONE's style requirements, including those for file naming. The PLOS ONE style templates can be found at

https://journals.plos.org/plosone/s/file?id=wjVg/PLOSOne_formatting_sample_main_body.pdf and

<https://journals.plos.org/plosone/s/file?id=ba62/PLOSOne_formatting_sample_title_authors_affiliations.pdf>

**Response: Done, manuscript presently meets PLOS ONE's style requirements, including those for file naming.**

[2.](https://journals.plos.org/plosone/s/file?id=ba62/PLOSOne_formatting_sample_title_authors_affiliations.pdf) Thank you for stating the following in the Acknowledgments Section of your manuscript:

"This project was supported by the Nigerian Institute of Medical Research who purchased reagents for the study."

We note that you have provided funding information that is not currently declared in your Funding Statement. However, funding information should not appear in the Acknowledgments section or other areas of your manuscript. We will only publish funding information present in the Funding Statement section of the online submission form.

Please remove any funding-related text from the manuscript and let us know how you would like to update your Funding Statement. Currently, your Funding Statement reads as follows:

"The authors received no specific funding for this work."

**Response: Funding statement removed from manuscript. Update my Funding Statement to read, “****The authors received no specific funding for this work”.**

 Please include your amended statements within your cover letter; we will change the online submission form on your behalf. **Response: Funding statement has been included in the cover letter.**

3. Your ethics statement should only appear in the Methods section of your manuscript. If your ethics statement is written in any section besides the Methods, please delete it from any other section. **Response: Ethics statement removed from other sections of the manuscript, left only in the Methods section.**

 4.Thank you for submitting the above manuscript to PLOS ONE. During our internal evaluation of the manuscript, we found significant text overlap between your submission and the following previously published works.

https://www.sciencedirect.com/science/article/pii/S1876034120304676?via%3Dihub
https://covidtestingproject.org/faq.html
https://ccpp19.org/healthcare_providers/virology/detecting_virus.html
https://journals.plos.org/plosone/article?id=10.1371%2Fjournal.pone.0233947

We would like to make you aware that copying extracts from previous publications, especially outside the methods section, word-for-word is unacceptable. In addition, the reproduction of text from published reports has implications for the copyright that may apply to the publications.

Please revise the manuscript to rephrase the duplicated text, cite your sources, and provide details as to how the current manuscript advances on previous work. Please note that further consideration is dependent on the submission of a manuscript that addresses these concerns about the overlap in text with published work.

We will carefully review your manuscript upon resubmission, so please ensure that your revision is thorough. **Response: All duplicated texts have been rephrased; sources have been cited.**

[Note: HTML markup is below. Please do not edit.]

Reviewers' comments:

Reviewer's Responses to Questions

**Comments to the Author**

1. Is the manuscript technically sound, and do the data support the conclusions?

The manuscript must describe a technically sound piece of scientific research with data that supports the conclusions. Experiments must have been conducted rigorously, with appropriate controls, replication, and sample sizes. The conclusions must be drawn appropriately based on the data presented.

Reviewer #1: Yes

Reviewer #2: Yes

Reviewer #3: Yes

  2. Has the statistical analysis been performed appropriately and rigorously?

 Reviewer #1: Yes

Reviewer #2: Yes

Reviewer #3: N/A

 3. Have the authors made all data underlying the findings in their manuscript fully available?

The [PLOS Data policy](http://www.plosone.org/static/policies.action#sharing) requires authors to make all data underlying the findings described in their manuscript fully available without restriction, with rare exception (please refer to the Data Availability Statement in the manuscript PDF file). The data should be provided as part of the manuscript or its supporting information, or deposited to a public repository. For example, in addition to summary statistics, the data points behind means, medians and variance measures should be available. If there are restrictions on publicly sharing data—e.g. participant privacy or use of data from a third party—those must be specified.

Reviewer #1: No

Reviewer #2: Yes

Reviewer #3: Yes

 4. Is the manuscript presented in an intelligible fashion and written in standard English?

PLOS ONE does not copyedit accepted manuscripts, so the language in submitted articles must be clear, correct, and unambiguous. Any typographical or grammatical errors should be corrected at revision, so please note any specific errors here.

Reviewer #1: Yes

Reviewer #2: Yes

Reviewer #3: Yes

 5. Review Comments to the Author

Please use the space provided to explain your answers to the questions above. You may also include additional comments for the author, including concerns about dual publication, research ethics, or publication ethics. (Please upload your review as an attachment if it exceeds 20,000 characters)

 Reviewer #1: Well written paper, easy to understand and clear.
Conclusions are also clear.

Line 66: it says the correct area—define the correct area as readers vary in understanding. **Response: Done. Statement changed.**

Table 1: Reflect how you calculated the sensitivity and specificity. Could label a, b, c, d. **Response: calculations have been reconstructed.**

How did you try to control or could not control for the refusers so there are no biases? How will authors know if the refusers may have actually tested positive or negative. What is the size of those who did not consent? This data will be helpful. or at least include in study limitations. **Response: Done, see Study limitations.**

Line 200: Not convinced that plasma will be helpful for detection though, can this be explained further? **Response: Statement changed to reflect that plasma will only be useful in antibody and antigen detection assays.**

Reviewer #2: This is an interesting study comparing the ability to detect SARS-COV-2 RNA within the circulation to gold standard nasal/oral swabs. The authors demonstrate that nasal/oral swabs are more accurate than using blood plasma.

The abstract/results needs to be amended; "There was no false positive recorded, but 119 (95.2%) false negatives were obtained by plasma". I believe that this should be changed to; "There was no false positive recorded, but 69 (55.2%) false negatives were obtained by plasma" The reason for this is that the authors were only able to detect 75/125 using swabs (gold standard) and only therefore know that these were true positive COVID-19 samples. The remaining 50/125 should be classed as negative. This would also alter the sensitivity & specificity calculations. This should also be updated in the results section.

**Response: Statement Change effected,** **however, it did not alter the sensitivity and specificity**.

Since it is now Feb 2021, the prevalence figures in the introduction need to be updated (data is from July 2020). **Response: Figures have been updated**

It is a shame that the authors do not have any data on symptom onset or longitudinal sampling. This would have greatly enhanced the impact of the manuscript. **Response: Unfortunately, no data on those. but I could attempt that in a subsequent research.**

Reviewer #3: Review
Low-level SARS-CoV-2 RNA detected in Plasma Samples from a cohort of Nigerians: implications for blood transfusion

This study assesses the specificity and sensitivity of the PCR tests performed in plasma samples in comparison with oral and nasal swab samples to detect Sars-CoV-2 infection. They found that plasma PCR had 95.2% of false negatives. Although the possibility of covid transmission through blood has yet to be determined, these results might have implications for blood transfusion testing requirements.


1. In the Study population section, please, include a table with the demographic (age, sex, etc) and clinical characteristics of the patients (number and type of symptoms). **Response: Done, please see Table 3 in the text.**

2. In page 6, line 120, it says that symptomatic and asymptomatic individuals were recruited; also, in page 9, line 181, it says that 20% of patients were asymptomatic, negative by plasma and positive by swab. However, in the Study population section (page 5, line 113) it says that the study criteria included presenting symptoms, such as fever, cough or breathing difficulty. Please, clarify whether there were asymptomatic patients and inclusion criteria. **Response: The statement has been clarified.**

3. What was the starting volume of plasma to extract RNA? Increasing the starting volume would probably increase the sensitivity of the test. **Response: The starting volume was 200 µL. That is true.**

4. In the description of the PCR method, please, include device and PCR parameters. **Response: The device has included and the PCR parameters and steps have been included.**

5. What was the minimum CT to consider a sample positive for Sars-CoV-2? **Response: 13.0**

6. If possible, it would be very interesting to look at the prevalence of antibodies against Sars-CoV-2 in the same cohort of patients. **Response: Not possible to carry out at this time.**

7. In Table 1, please, add statistical analysis and p-values, and describe the statistics used in the Methods section. **Response: Done.**

8. In page 8, line 166, the Cts of the plasma positive samples are included; please, add also the Cts of the same samples that were obtained by nasal/oral swab to better compare both methods. **Response: Done in Table 2.**

9. In Discussion, page 9, line 196, a preprint is cited. In general, citation of non-peer-reviewed articles should be avoided. If cited, clearly indicate in the main text that is a pre-print and has not been peer-reviewed. **Response:** **Done, preprint removed.**

10. At least another paper on the same subject has been published (J Clin Virol. 2020 Dec. Low prevalence of SARS-CoV-2 in plasma of COVID-19 patients presenting to the emergency department). Please, discuss. **Response: Paper discussed and cited.**

11. Discussion of reports showing covid transmission from mother to newborn might be relevant for blood transmission. **Response: such discussion not included.**


Minor corrections:

1. Substitute the term “clients” by “patients”, “individuals” or “participants”.
2. Page 3, line 51: It says: “The virus was originated in bats”. This has yet to be proved. Better to say “likely”. **Response: done**

 6. PLOS authors have the option to publish the peer review history of their article ([what does this mean?](https://journals.plos.org/plosone/s/editorial-and-peer-review-process#loc-peer-review-history)). If published, this will include your full peer review and any attached files.


If you choose “no”, your identity will remain anonymous but your review may still be made public.


**Do you want your identity to be public for this peer review?** For information about this choice, including consent withdrawal, please see our [Privacy Policy](https://www.plos.org/privacy-policy).

Reviewer #1: No

Reviewer #2: No

Reviewer #3: No

[NOTE: If reviewer comments were submitted as an attachment file, they will be attached to this email and accessible via the submission site. Please log into your account, locate the manuscript record, and check for the action link "View Attachments". If this link does not appear, there are no attachment files.]

While revising your submission, please upload your figure files to the Preflight Analysis and Conversion Engine (PACE) digital diagnostic tool, <https://pacev2.apexcovantage.com/>. PACE helps ensure that figures meet PLOS requirements. To use PACE, you must first register as a user. Registration is free. Then, login and navigate to the UPLOAD tab, where you will find detailed instructions on how to use the tool. If you encounter any issues or have any questions when using PACE, please email PLOS at [figures@plos.org](mailto:figures@plos.org). Please note that Supporting Information files do not need this step.

*In compliance with data protection regulations, you may request that we remove your personal registration details at any time.* [*(Remove my information/details)*](https://www.editorialmanager.com/pone/login.asp?a=r)*. Please contact the publication office if you have any questions.*
